# Evaluation of *Borrelia burgdorferi* BbHtrA Protease as a Vaccine Candidate for Lyme Borreliosis in Mice

**DOI:** 10.1371/journal.pone.0128868

**Published:** 2015-06-15

**Authors:** Amy J. Ullmann, Theresa M. Russell, Marc C. Dolan, Martin Williams, Andrias Hojgaard, Zachary P. Weiner, Barbara J. B. Johnson

**Affiliations:** Bacterial Diseases Branch, Division of Vector Borne Diseases, Centers for Disease Control and Prevention, Fort Collins, CO, United States of America; University of Toledo School of Medicine, UNITED STATES

## Abstract

*Borrelia burgdorferi* synthesizes an HtrA protease (BbHtrA) which is a surface-exposed, conserved protein within Lyme disease spirochetes with activity toward CheX and BmpD of *Borrelia* spp, as well as aggrecan, fibronectin and proteoglycans found in skin, joints and neural tissues of vertebrates. An antibody response against BbHtrA is observed in Lyme disease patients and in experimentally infected laboratory mice and rabbits. Given the surface location of BbHtrA on *B*. *burgdorferi* and its ability to elicit an antibody response in infected hosts, we explored recombinant BbHtrA as a potential vaccine candidate in a mouse model of tick-transmitted Lyme disease. We immunized mice with two forms of BbHtrA: the proteolytically active native form and BbHtrA ablated of activity by a serine to alanine mutation at amino acid 226 (BbHtrA^S226A^). Although inoculation with either BbHtrA or BbHtrA^S226A^ produced high-titer antibody responses in C3H/HeJ mice, neither antigen was successful in protecting mice from *B*. *burgdorferi* challenge. These results indicate that the search for novel vaccine candidates against Lyme borreliosis remains a challenge.

## Introduction

Lyme borreliosis is the most commonly reported tick-borne disease in the United States with approximately 35,307 cases in 2013 [1] and the disease is also highly prevalent in Europe and Asia with 65,000 cases reported in the former in 2011 [2, 3]. Many cases are unreported; the true burden of diagnosed Lyme disease in the United States has been estimated to be about 300,000 cases per year [4]. When properly diagnosed, Lyme disease can be effectively treated with antibiotic therapy. Some patients, however, go undiagnosed, or develop post-treatment sequelae, such as antibiotic refractory arthritis indicating a need for improved treatments and better preventive methods. Currently, prevention of Lyme disease is limited to personal protective measures against tick bites since no vaccine is commercially available [5].

The enzootic cycle of the causative agent of Lyme disease, *Borrelia burgdorferi sensu stricto*, between tick vectors and vertebrate hosts is complex. *Borrelia* spirochetes have mechanisms for differentially expressing gene products in response to temperature, pH and osmolarity in order to survive in the diverse milieus encountered in either ticks or mammals [6, 7]. For example, outer surface protein A (OspA) is expressed by *B*. *burgdorferi* in a tick vector, *Ixodes scapularis*, but is rarely produced in vertebrate hosts early in infection [8]. OspA was the target antigen for the LYMErix vaccine with activity based upon the ability of host antibodies against OspA to bind and neutralize *B*. *burgdorferi* in the midgut of an *I*. *scapularis* tick, thereby blocking transmission to the same host [9–11]. A biologic limitation of LYMErix, which was withdrawn from the market in 2002, was that OspA is expressed by *B*. *burgdorferi* in unfed ticks but is down-regulated during tick feeding. Hosts are exposed to very little OspA at the time of tick bite, precluding a natural boost to the immune response and requiring an annual booster shot of the vaccine to maintain a high titer of antibody for full protection [12]. Given the limitations of this and other antigens for vaccination, attempts to identify alternative vaccine candidates from surface-expressed proteins of *B*. *burgdorferi* continue.

*B*. *burgdorferi* High Temperature Requirement A (BbHtrA) was recently described as a surface-exposed and conserved protease within Lyme disease spirochetes [13, 14]. Proteases are critical proteins throughout the animal kingdom as they function in protective and regulatory roles for other proteins in the cell cycle [15]. BbHtrA has activity toward CheX, which is involved in spirochete motility, and BmpD, an outer membrane protein, of *B*. *burgdorferi*, as well as aggrecan, fibronectin and proteoglycans found in vertebrate skin, joint and neural tissue [13, 14, 16]. The degradative activity of BbHtrA targeting extracellular matrix proteins of vertebrates *in vitro* suggests a role in bacterial dissemination within the host for establishment of infection [16].

An antibody response to BbHtrA is observed in Lyme disease patients as well as in experimentally infected laboratory mice and rabbits [13]. Precedent exists for HtrA proteins as protective immunogens in other disease models including *Haemophilus influenzae* [17], *Orientia tsutsugamushi* [18], and *Chlamydia muridarum* [19]. Given the exposure of BbHtrA on the surface of *Borrelia* and its ability to elicit an immune response in infected hosts, we explored recombinant BbHtrA as potential vaccine candidate in a mouse model of tick-transmitted Lyme disease. Two forms of BbHtrA were evaluated: a mutant protease with ablated activity due to a substitution of alanine for serine at amino acid 226 (BbHtrA^S226A^) and the wild type protease with intact proteolytic capacity.

## Methods and Materials

### Ethics statement

The Division of Vector Borne Diseases, NCEZID, CDC, Animal Care and Use Committee approved study protocol #14–002 for vaccinating mice, feeding of ticks on mice, infecting mice with spirochetes, and the isolation tissues from mice. All work in our study was conducted adhering to the institution’s guidelines for animal husbandry, and followed the guidelines and basic principals in the Public Health Service Policy on Humane Care and Use of Laboratory Animals, and the Guide for the Care and Use of Laboratory Animals, United States Institute of Laboratory Animal Resources, National Research Council.

### Immunization and challenge of mice

Recombinant BbHtrA and BbHtrA^S226A^ were previously generated [14]. BbHtrA^S226A:^ 18 μg in injection buffer (IB) (50 mM HEPES, 300 mM NaCl_2_) was adsorbed to Imject Alum (Pierce, Rockford, IL) per manufacturer’s instructions. Eight mice were injected with IB + Imject Alum and 8 were injected with BbHtrA + Imject alum. Mice were boosted at days 21 and 42. Blood was collected for serology from the facial artery/vein plexus at days 0, 29, 49 and 91. Three *B*. *burgdorferi* (laboratory strain B31) infected *I*. *scapularis* nymphal ticks were placed on each mouse at day 63 post-immunization and allowed to feed to repletion. Replete ticks were collected and cultured in BSK-II media or processed for TaqMan PCR with endpoint detection as previously described [20] to confirm infection status. At day 91, mice were sacrificed and ear, heart and bladder were collected and cultured in BSK-II medium for growth of *B*. *burgdorferi* and observed by dark field microscopy to determine infection status. BbHtrA: 14.6 μg in injection buffer was adsorbed to Imject Alum or emulsified with TiterMax Gold (Sigma, St. Louis, MO) per manufacturer’s instructions. Six mice were injected with IB + Imject Alum, 6 mice were injected with IB + TiterMax Gold, 9 mice were injected with BbHtrA + Imject Alum, and 9 mice were injected with BbHtrA + TiterMax Gold. Mice were boosted at day 21 and 42. One mouse in the IB + TiterMax Gold group and one mouse in the BbHtrA/TiterMax group died while on study. Blood was collected for serology from the facial artery/vein plexus at days 0, 29, 49, 70 and 104. Four *B*. *burgdorferi* B31 infected *I*. *scapularis* nymphal ticks were placed on each mouse at day 83 and allowed to feed to repletion. At day 104, mice were sacrificed and ear, heart and bladder were collected and placed into BSK-II medium to determine infection status.

### Recombinant BbHtrA-mouse serum ELISA

Microtiter plates (Immulon 2HB, Thermo Scientific) were coated overnight at 4°C with 150 ng of recombinant BbHtrA or BbHtrA^S226A^ in 100 μl carbonate coating buffer (90 mM NaHCO_3_ and 60 mM Na_2_CO_3_, pH 9.6). All washing steps were performed with 13 mM Tris HCl, 3 mM Tris base (pH 7.4), 140 mM NaCl, 2.7 mM KCl, and 0.05% Tween 20 (TBS-T) utilizing an automated plate washer (SkanWasher 300, Skatron) with 5 cycles of 500 μl TBS-T. BbHtrA-coated plates were washed and incubated for 2 h at room temperature (RT) with 300 μl blocking buffer (Starting Block, Pierce). Blocked, duplicate wells, were incubated for 1 h at RT with 100 μL of each serum sample 2-fold serially diluted starting at a 1:400 dilution in blocking buffer. After washing, plates were incubated for 30 min at 37°C with secondary antibody (0.1 μg/ml diluted in blocking buffer, alkaline phosphatase-conjugated goat anti-mouse IgG, KPL). After two final washes, 100 μL of alkaline phosphatase substrate (p-nitrophenyl phosphate; Sigma N9389, 1 mg/mL diluted in 23 mM NaHCO3, 25 mM Na_2_CO_3_, and 0.1 M MgCl_2_, pH 9.8) was added to each well, and the optical density at 405 nm was measured using a Bio-Tek EL808 shaking ELISA plate reader and Gen5 software (Bio-Tek Instruments). Baseline was established by subtracting the 1:400 dilution values from pre-challenge, adjuvant only serum. Reciprocal 90% end-point titers were defined as the serum dilution at which there was a 90% reduction in signal from the starting 1:400 dilution.

### Native BbHtrA-mouse serum IgG immunoblots

Commercially available *B*. *burgdorferi* lysate strips containing in vitro-cultivated strain B31 (*Borrelia* B31 IgG ViraBlots, ViraMed, Planegg, Germany) were incubated for 1 h at RT with 100 μL of each serum sample diluted 1:100 in blocking buffer. Strips were washed with TBS-T 3 times for 5 min each and incubated for 45 min at RT with secondary antibody (0.02 μg/ml diluted in blocking buffer, alkaline phosphatase-conjugated goat anti-mouse, KPL). After 4 washes, strips were incubated for 20 min with precipitating AP substrate (BioRad 170–6432).

### Challenge of mice with *B*. *burgdorferi* strain B31 by tick-bite

5–6 week-old female C3H/HeJ mice were purchased from the Jackson Laboratories (Bar Harbor, ME) and housed in HEPA-filtered cages. Laboratory-reared nymphal *I*. *scapularis* ticks were infected with *B*. *burgdorferi* strain B31 (infection rate, ≥90%) as previously described [21]. Mice were anesthetized by inhalation of isoflurane and 3–4 nymphal ticks were placed dorsally between the scapulae and allowed to feed to repletion. Replete ticks were recovered and either processed for DNA extraction and PCR confirmation of *B*. *burgdorferi* infection [20] or cultured in BSK-II medium supplemented with antibiotics and fungizone to confirm infection. The mice were assayed for infection at 28 days post challenge by serology and culture of ear, heart and bladder at necropsy in BSK-II medium supplemented with antibiotics and fungizone as previously described [22]. The Animal Care and Use Committee at the Division of Vector Borne Diseases, CDC, Fort Collins, CO approved all experimental protocols involving mice.

### Statistical analysis

Significant differences between experimental groups and control groups were determined using Fisher’s Exact Test of Probability. P-values were determined for each sample. A p-value of < 0.05 was considered to be significant

## Results

**Fig 1:**
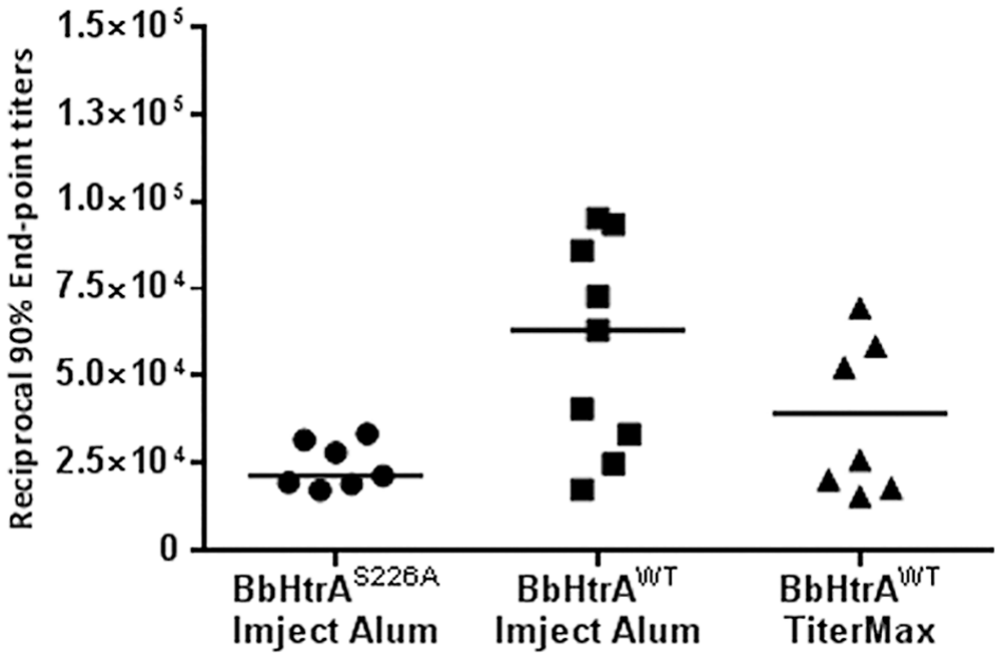
Comparison of pre-challenge reciprocal 90% end-point titers for mice vaccinated with BbHtrA^S226A^ or BbHtrA^WT^ in Imject Alum or TiterMax adjuvants. 0 corresponds to baseline established from adjuvant-only, pre-challenge serum at day 70. Bars represent median values.

**Fig 2:**
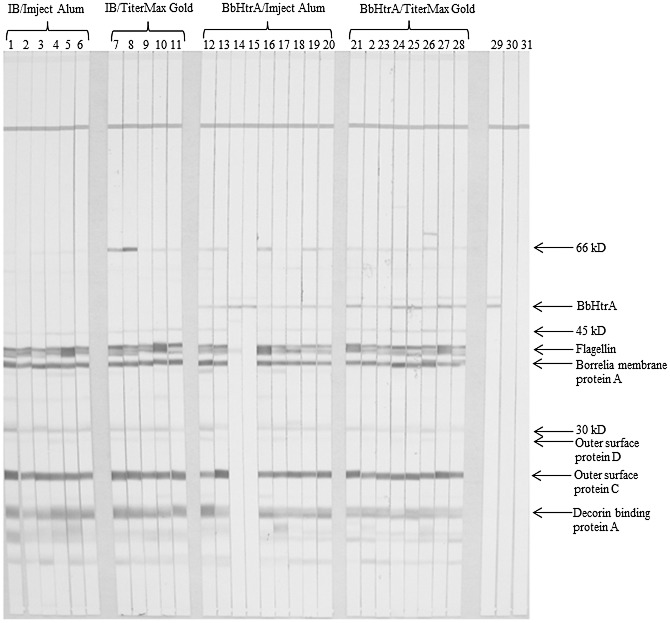
IgG immunoblots probed with serum from BbHtrA immunized mice. Serum is considered positive for antibodies to *B*. *burgdorferi* when ≥ 5 bands are present. Lanes 1–6 are Injection Buffer (IB)/Imject Alum, 7–11 are IB + TiterMax Gold, 12–20 are BbHtrA + Imject Alum, and 21–28 are BbHtrA + TiterMax Gold. Lane 29 was probed with mouse monoclonal anti-BbHtrA antibody. Lanes 30 and 31 was probed with goat anti-human and goat anti-mouse secondary antibodies alone. * indicates recognition of native BbHtrA by serum of immunized mice that did not seroconvert after challenge with infected ticks.

In the first experiment, mice were immunized with recombinant BbHtrA^S226A^ with Imject Alum as the adjuvant, followed by two booster injections. Prior to challenge with *B*. *burgdorferi* infected *I*. *scapularis* ticks, mice were bled at day 49 and determined by ELISA to have seroconverted. 90% end-point titers were observed at serum dilutions of less than 1:40,000 in all mice vaccinated with BbHtrA^S226A^ (median 21,570, Fig 1A, column 1). No adverse effects were observed in any of the mice. Mice were sacrificed three weeks post-challenge and serology as well as culture of ear, heart and bladder was performed. The observed antibody response was inadequate to prevent *B*. *burgdorferi* infection by tick bite (Table 1 and Fig 2). With the exception of one mouse in the BbHtrA^S226A^ control group (injection buffer/Imject Alum), all mice were confirmed to have been fed upon by *B*. *burgdorferi* infected *I*. *scapularis* ticks with tick infection confirmed by PCR (Table 1).

**Table 1:** Infection status of vaccinated mice after challenge with *B*. *burgdorferi* infected ticks.

Experimental Group	No. of mice positive by culture	No. of mice positive by Virablot analysis	No. of infected mice/total no. of mice challenged^a^	Fisher’s Exact Probablility Test p-value
BbHtrA^S226A^ control (Injection buffer/Imject Alum)	6/7	6/7	6/7^b^	
BbHtrA^S226A^ / Imject Alum	7/7	7/7	7/7	0.4999
BbHtrA control (Injection buffer/Imject Alum)	6/6	6/6	6/6	
BbHtrA control (Injection buffer/TiterMax Gold)	6/6	6/6	6/6	
BbHtrA/Imject Alum	7/9	7/9	7/9^c^	0.3429
BbHtrA/TiterMax Gold	9/9	9/9	9/9	0.9999

^a^Infection was assessed by culture of ear, heart and bladder as well as serology.

^b^No ticks were recovered from the one mouse that was uninfected in this group.

^c^Only a single infected tick was recovered from one uninfected mouse and no infected ticks were recovered from the second uninfected mouse.

For experiment two, mice were given an initial inoculation of recombinant wild type BbHtrA with either Imject Alum or TiterMax Gold as the adjuvant. Antibody titers to BbHtrA were examined by ELISA in 4 mice from each group at day 49 and six of eight mice had end-point titers of less than 1:45,000 (median 27,092, data not shown). In an effort to increase the response to BbHtrA^WT^, all mice were boosted a third time on day 63. No adverse effects were seen in any of the immunized mice. At day 70, all mice achieved pre-challenge antibody titers greater than 1:15,000 and 9 of 17 mice achieved end-point titers at dilutions greater than 1:50,000 (Fig 1A, columns 2 and 3). Higher titers were observed in the group vaccinated with Imject Alum as the adjuvant (median 63,200, Fig 1, column 2) versus the group vaccinated with TiterMax as the adjuvant at day 70 (median 39,344, Fig 1 column 3). Mice were sacrificed three weeks post tick challenge and serology demonstrated that 16 of 18 BbHtrA-vaccinated mice became infected by *B*. *burgdorferi* when challenged by infected ticks (Fig 2).

Two mice in the BbHtrA/Imject Alum group did not become infected as evidenced by the absence of serodiagnostic antibodies (Fig 2, lanes 15 and 16) and negative cultures of ears, hearts, and bladders (Table 1). At the end of the experiment, the infection status of the challenge ticks was confirmed by culture. At least one infected tick was recovered from 28 of 29 mice (data not shown). Of the two uninfected mice, only one infected tick was recovered from one while no ticks were recovered from the other mouse, while all other mice had confirmed infected tick bite (Table 1).

## Discussion

We hypothesized that BbHtrA would be a protective antigen against tick transmitted *B*. *burgdorferi* infection based on its biophysical properties and the precedents for use of HtrAs as vaccines for other bacterial infections [17, 19, 23]. The protease is surface exposed, elicits a strong antibody response in infected hosts, and stimulates inflammatory responses *in vitro* which led us to examine BbHtrA as a potential vaccine candidate. It has been demonstrated by several groups that cultured *B*. *burgdorferi* is not the most appropriate vehicle for infectious challenges, as it does not exhibit the same protein expression patterns as organisms in ticks that adapt their surface structures to the tick feeding environment. We therefore challenged our immunized mice by the more rigorous method of infectious tick bite. Results from this study demonstrated that immunization with either proteolytically inactive or wild-type BbHtrA was not protective against *B*. *burgdorferi* challenge by feeding *I*. *scapularis*.

Initially, we chose to explore BbHtrA ablated of protease activity to prevent potential adverse effects from injecting an active protease knowing that polyclonal antibodies developed against wild-type BbHtrA also reacted to BbHtrA^S226A^. Although the host generated an antibody response against BbHtrA^S226A^, none of the mice were protected from *B*. *burgdorferi* infection. To ensure that the lack of vaccine efficacy observed with BbHtrA^S226A^ was due to inherent non-protective properties of the protein, we repeated the vaccination protocol with wild-type BbHtrA. We also chose to use a second adjuvant, TiterMax Gold, to test for host enhancement of antibody titer, as often adjuvant choice is determined empirically. The use of two different adjuvants also had the advantage of intentionally stimulating a Th2 response with Imject Alum and a Th1 response with TiterMax Gold [24–26]. In spite of these additional measures, 16 of 18 immunized mice became infected (Fig 2). Although the optimal concentrations of protease utilized for immunizations were not determined by a titration series, the doses administered were consistent with those which demonstrated partial protection in vaccine trials with HtrA proteases from other organisms [17, 19, 23, 27].

Two of the mice in the Imject Alum BbHtrA^WT^ vaccination group remained uninfected as assessed by serology and by culture of ear, heart, and bladder for *B*. *burgdorferi*. No ticks were recovered from one of the uninfected mice and the other mouse had only one infected tick recovered from the feeding. Additionally, the end-point titers for these mice were observed at dilutions greater than 60,000. Seven of seventeen vaccinated animals in this experiment achieved end-point titers equal to or greater than those observed for the two uninfected mice. Thus, antibody titers cannot explain the uninfected status of the two mice; it seems likely that the mice did not receive an infectious dose of *B*. *burgdorferi*.

In conclusion, although BbHtrA is immunogenic in mice, it did not prove to be an effective vaccine candidate. Elucidating the important immunogenic factors of *B*. *burgdorferi* continues to be a priority as we work toward developing preventive tools for controlling Lyme disease.
